# Transcriptome analysis of *Glomus mosseae*/*Medicago sativa* mycorrhiza on atrazine stress

**DOI:** 10.1038/srep20245

**Published:** 2016-02-02

**Authors:** Fuqiang Song, Jize Li, Xiaoxu Fan, Quan Zhang, Wei Chang, Fengshan Yang, Gui Geng

**Affiliations:** 1 Bio-ecological Restoration, School of Life Science, Heilongjiang University, Harbin, 74 Xuefu Road Heilongjiang 150080, China; 2 Department of Biology, Washington University at St Louis, Saint Louis, 1 Brookings Drive MO 63130, USA

## Abstract

Arbuscular mycorrhizal fungi (AMF) protect host plants against diverse biotic and abiotic stresses, and promote biodegradation of various contaminants. In this study effect of *Glomus mosseae/Medicago sativa* mycorrhiza on atrazine degradation was investigated. It was observed that the atrazine degradation rates with any addition level in mycorrhizal treatments were all significantly higher than those in non- mycorrhizal treatments. When atrazine was applied at 20 mg kg^−1^, the removal efficiency was up to 74.65%. Therefore, *G. mosseae* can be considered as ideal inhabitants of technical installations to facilitate phytoremediation. Furthermore, a total of 10.4 Gb was used for de novo transcriptome assembly, resulting in a comprehensive data set for the identification of genes corresponding to atrazine stress in the AM association. After comparative analysis with edgeR, a total of 2,060 differential expressed genes were identified, including 570 up-regulated genes and 1490 down-regulated genes. After excluding ‘function unknown’ and ‘general function predictions only’ genes, 172 up-regulated genes were obtained. The differentially expressed genes in AM association with and without atrazine stress were associated with molecular processes/other proteins, zinc finger protein, intracellular/extracellular enzymes, structural proteins, anti-stress/anti-disease protein, electron transport-related protein, and plant growth associated protein. Our results not only prove AMF has important ecological significance on atrazine degradation but also provide evidence for the molecular mechanisms of atrazine degradation by AMF.

Atrazine is frequently used alone or in combination with other herbicides for combating grassy and broadleaf weeds in *Zea mays* and *Triticum aestivum* production, with 70,000–90,000 tons applied annually in the world^1^. So far it is an extensively used herbicide in the Northeast China, the largest grain production center. However, atrazine and its degradation products, mainly desethylatrazine and deisopropylatrazine, have been suggested to inflict damage to the central nervous, the endocrine and immune systems^2^. Due to its large-scale application and long half-life, atrazine has high potential to contaminated aquifers, groundwater, rain water and drinking water sources^3^. Therefore, the study on bioremediation strategies for atrazine polluted environments has become a hotspot due to its cost efficiency and environmental friendliness.

Atrazine degradation efficiency and growth characteristics of a wide range of bacteria have been reported worldwide^2^. These successful microbial remediation in a laboratory setting is often more difficult to achieve *in situ* of contaminated sites^4^. In addition, researchers have endeavored to use microbes to facilitate phytoremediation including biodegradative bacteria, plant growth-promoting bacteria and arbuscular mycorrhizal fungi^5^. The arbuscular mycorrhizal fungi (AMF), belonging to the phylum Glomeromycota, form mutualistic associations with most vascular plant species. Once an arbuscular mycorrhiza (AM) association develop, mycorrhizal plants take up water and mineral nutrients, such as phosphorus (P), nitrogen (N), and trace elements more efficiently than non-mycorrhizal plants^6^,^7^. Meanwhile, AMF protect host plants against diverse biotic and abiotic stresses^8^. Most previous studies have found that AM fungi have positive effects on the dissipation of organic contaminants such as atrazine. Huang *et al*. observed that atrazine dissipation in soil was enhanced by AM association^9^,^10^. Regardless, how strong maybe the affect and what the contribution of these pollutants to the mycorrhizosperic processes remains mostly uncertain.

In our previous studies, we observed that *Glomus mosseae,* the dominant species in Heilongjiang Province of China, associated with *Sorghum bicolor* could reduce a maximum 91.6% of atrazine significantly^11^. Recently, developed high-throughput messenger RNA (mRNA) sequencing (RNA-Seq) technology has been extensively employed to study genome-wide gene expression in living organisms^12^. In this study, *G. mosseae/Medicago sativa*, one of atrazine-sensitive plant mycorrhiza with and without atrazine stress was investigated. A total of 10.4 Gb was used for *de novo* transcriptome assembly, resulting in a comprehensive data set for the identification of genes corresponding to atrazine stress in the AM association. The objective of this work was to prove AMF has important ecological significance on atrazine degradation^9^,^10^,^11^, and provide evidence for the molecular mechanisms of atrazine degradation by AMF^27^,^28^.

## Results

### Colonization of roots by *G. mosseae*

**Figure 1:**
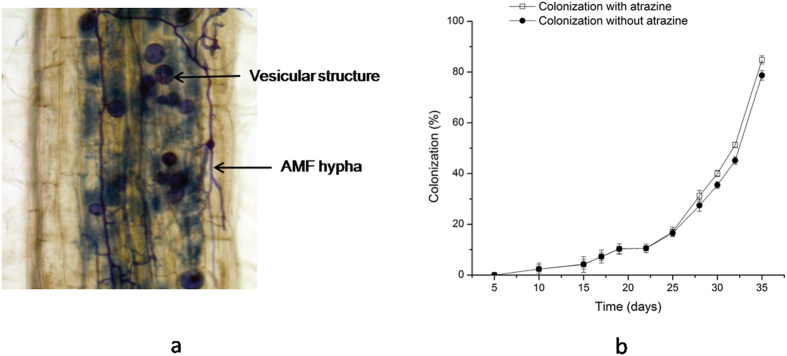
(**a**) Images of AMF colonization, (**b**) Changes of AMF colonization rates with time.

Roots of inoculated *M. sativa* were extensively colonized by *G. mosseae*. No mycorrhizal colonization was observed in control plants. Excellent symbiotic relationship between *G. mosseae* and *M. sativa* was established with the formation of vesicular structures and AMF hypha (Fig. 1a). The colonization rate reached 85.67% under the stress of atrazine, and 75% without the stress of atrazine after 35 d (Fig. 1b).

### Degradation rate of atrazine

**Figure 2:**
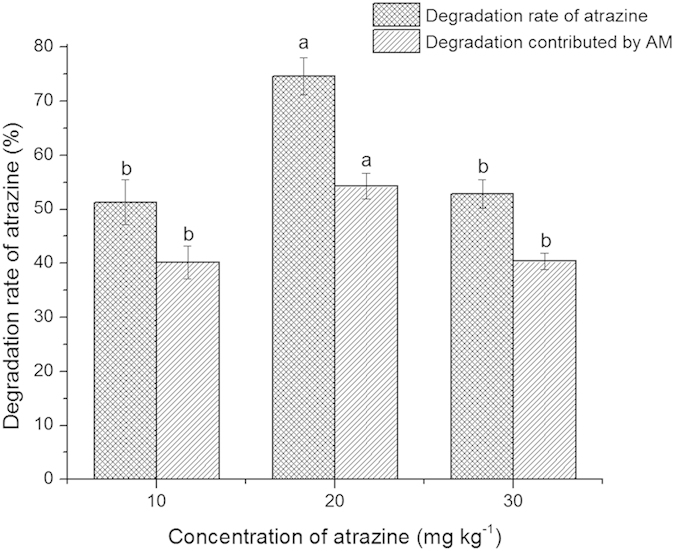
Degradation rate of atrazine in mycorrhizal treatment and its degradation rate contributed by AM. The degradation rate contributed by AM is the difference rate between mycorrhizal and non-mycorrhizal treatments. Different letters indicate significantly different values at *P* < 0.05. Error bars represent the standard error of mean of three replicates (n = 3).

Compared with the initial concentrations, the atrazine concentration of Compartment A in inoculated treatments decreased from 51.29% to 74.65% (Fig. 2), those 11.11% to 12.47% in non-inoculated controls (data not shown). Inoculation significantly decreased the amount of residual atrazine in soil (*P* < 0.05). The dissipation of atrazine in non-mycorrhizal treatments was the results of host plants uptake and other environmental degradations. The differences decrease of atrazine between the mycorrhizal and control treatments attributes to AM in any atrazine addition level. When the atrazine was applied at 20 mg kg^−1^, degradation rate and its contributed by AM were both significantly higher than those in other addition levels.

### Library construction

**Figure 3:**
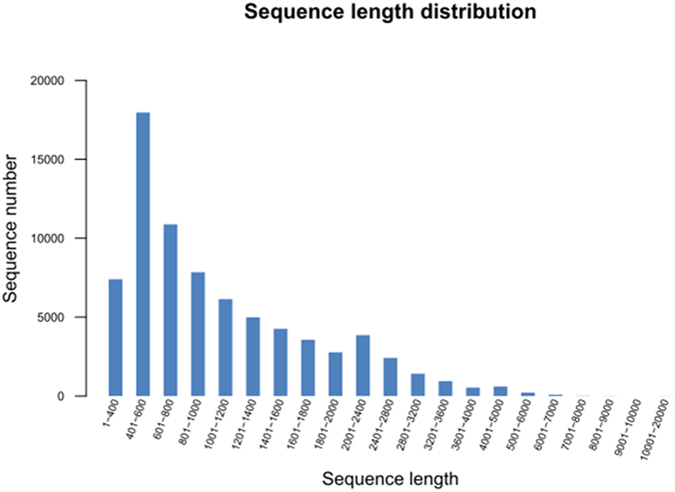
The diagram of assembly results and length distribution.

A cDNA library was obtained from an equal mixture of RNA isolated from the above two AM roots (with 20 mg kg^−1^ atrazine and without atrazine) and then used for Illumina sequencing. Using 101 bp pair-end sequencing based on Illumina sequencing approach, a total of 103,134,502 reads were obtained and then *do novo* assembled using Trinity Software. The longest assembled sequences containing blocks of unknown bases (Ns) were called contigs. A total of 75,957 contigs from 351 to 14643 bp were assembled with a mean size of 1132.66 bp (Table 1). The size of the sequence and distribution to assemble can be seen in Fig. 3.

### Annotation of predicted proteins

**Figure 4:**
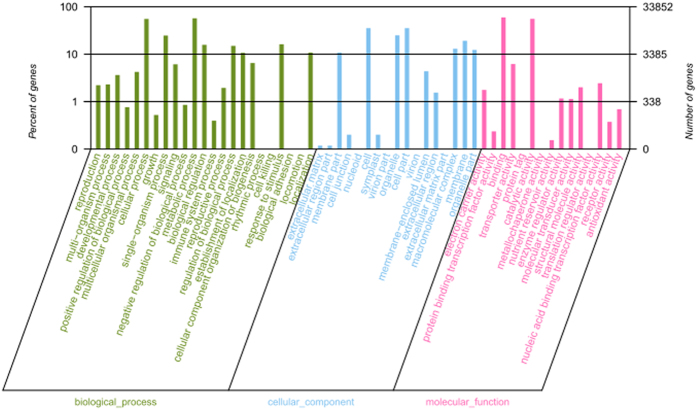
Gene ontology classifications of the assembled unisequences. The results are summarized in three main categories: biological process, cellular component and molecular function. In total, 45807 unisequences with BLAST matches to known proteins were assigned to gene ontology groups.

**Figure 5:**
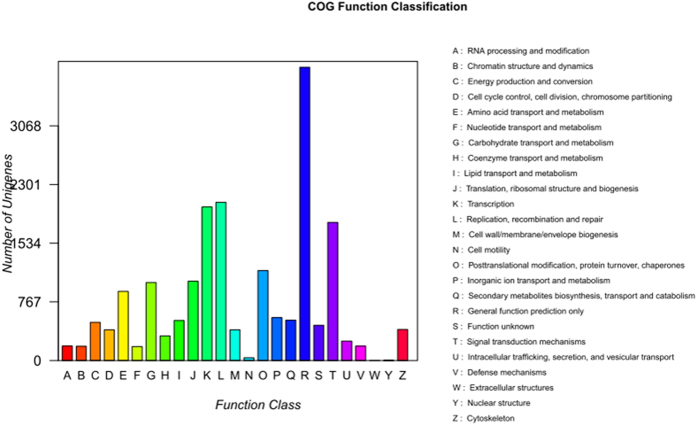
The result of COG function classification.

Sequence similarity search was conducted with non-redundant (nr) database, UniProtKB/Swiss-Prot (SwissProt), Gene Ontology (GO), Clusters of Orthologous Groups (COG) and the Kyoto Encyclopedia of Genes and Genomes (KEGG) database. GO assignments were utilized to classify the predicted functions of *M. sativa* mycorrhizal genes. Based on sequence homology, 45,807 unisequences were categorized into 55 functional groups (Fig. 4). In each of the three main categories (biological process, cellular component, and molecular function) of the GO classification, the metabolic process, diverse cellular activities and catalytic activity terms were dominant, respectively. In addition, all clusters were submitted to a search against the Cluster of COG database for functional prediction and classification. In total, 24,104 sequences showing Nrhits were assigned to COG classifications. Among the 25 COG categories, the cluster for ‘General function prediction’ represents the largest group (3834; 15.88%) followed by ‘Replication, recombination and repair’ (3728; 10.0%) and ‘Transcription’ (2068; 8.57%), with the following nuclear structure (5; 0.002%), and Cell motility (36; 0.15%) being the smallest groups (Fig. 5).

### Differential expression analysis

**Figure 6:**
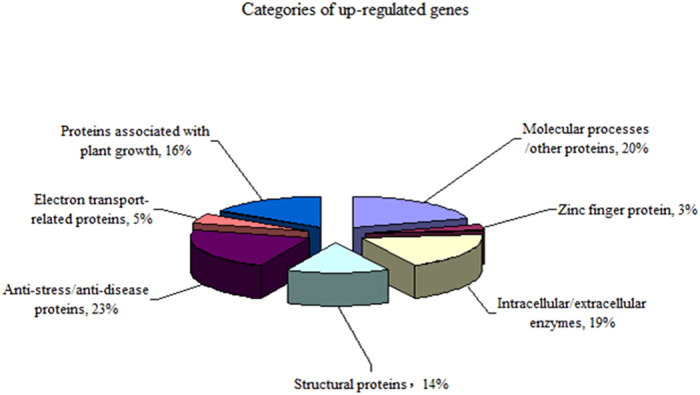
The function categories of up-regulated genes.

Two samples for sequencing were collected from AM under 20 mg kg^−1^ atrazine addition and without treatments respectively. After comparative analysis with edgeR, a total of 2,060 differential expressed genes were identified, including 570 up-regulated genes and 1490 down-regulated genes (removed from further analysis in this study). Excluding ‘function unknown’ and ‘general function predictions only’ genes, 172 functionally annotated genes were found in 570 up-regulated genes. We divide these genes into 7 categories according to their functions (Fig. 6), including anti-stress/anti-disease protein, molecular processes/other proteins, zinc finger protein, intracellular/extracellular enzymes, structural proteins, electron transport-related protein and protein associated with plant growth (Tables 2 and 3).

### The verification results of RT-PCR

**Figure 7:**
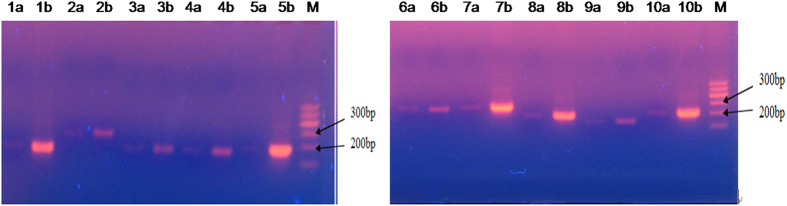
1% agarose gel electrophoresis figure of RT-PCR.

Comp441_c0, comp80087_c0, comp81470_c0, comp57797_c0, comp29448_c0, comp74170_c0, comp13165_c0, comp63527_c0, comp50160_c0, and comp279364_c0 were selected in RT-PCR. Above genes corresponded to lane 1a to 10b, respectively “a” and “b” indicated one gene without and with atrazine addition treatments. As shown in Fig. 7, genes expressions in atrazine addition treatments were higher than those in without atrazine addition.

## Discussion

In recent years, bioremediation of atrazine has become a hot topic. However, few researches on bioremediation by plant associated with fungi, particularly AMF in atrazine contaminated soil were reported. When 20 mg kg^−1^ of atrazine added, the degradation rate reached to 74.65% as compared to control showing 15.94% respectively. Results revealed a significant decrease in the residual atrazine concentration might be due to the AMF effect which is persistent with previous findings. When the application rate was 5.0 mg kg^−1^, the removal efficiency of atrazine by *G. etunicatum/Zea mays* mycorrhiza was up to 77.3%^10^. In addition, several studies have recently demonstrated beneficial effects of AMF on the biodegradation of other organic pollutants, including polycyclic aromatic hydrocarbons (PAHs) by *Lolium multiflorum* associated with *G. mosseae*^13^, dissipation of polychlorinated biphenyis pollution (PCB) by *Cucurbita pepo* with *G. mosseae*^14^, and phytoremediation of heavy metals (Pd, Cu, Zn, As) inoculated with *G. mosseae*^15^,^16^,^17^. Therefore, arbuscular mycorrhizal fungi, especially *G. mosseae* can be considered as ideal inhabitants of technical installations for phytoremediation.

In transcriptome sequencing, high-level expression of some sequences, including encoding zinc finger protein, anti-stress/anti-disease proteins, electron transport-related proteins, and laccase and glutathione peroxidase under the stress of atrazine were obtained. Among these genes, laccase and glutathione peroxidase have been known to play a major role in the degradation of atrazine. By analyzing the differentially expressed genes in AM association with and without atrazine stress, 172 up-regulated genes were identified which includes 5 genes encoding for zinc finger proteins and 39 genes for anti-stress/anti-disease proteins.

Increasing evidences have revealed that zinc finger proteins (ZFPs), a well conserved and large family in many plant species participated in the regulation of plant growth, developmental processes and resistance mechanism for various biotic and abiotic stresses^18^. Cys2His2 (C2H2)-type ZFPs belong to a major family of transcription factors that implicated in different cellular processes involved in the plant development and responses to cold, drought and other abiotic stress^19^,^20^. Gaude *et al*. found that the transcription factors encoded C2H2-type ZFPs most strongly induced in arbuscule-containing and non-colonized cortical cells of *M. truncatula*/*G. intraradices* mycorrhizal roots^21^. A total of 34 *Medicago* Cysteine3Histidine (CCCH) zinc finger genes were found to be unevenly distributed on eight chromosomes and displayed different expression levels in response to various stress conditions^22^. Overexpression of ZFPs could enhance resistance to fungal disease including *Rhizoctonia solani, Puccinia striiformis* f. sp. *tritici* and *Magnaporthe oryzae*^23^,^24^,^25^. However, high-level expression of sequences encoding zinc finger proteins may be distributed to double xenobiotic stress, *G. mosseae* and atrazine. The main function of the Zinc finger proteins is to trigger the expression of down-relgulated genes in response to stress. High level expression of zinc finger proteins in symbiotic roots under the stress of atrazine not only increases the resistance of adversity, but might be directly involved in atrazine degradation.

High level expression genes in 39 anti-stress/anti-disease proteins, such as NAC transcription factors and ethylene response transcription factor, might also play an important role in atrazine resistance. Wang *et al*. found that NAC transcription factors were involved in biotic and abiotic stress response in plant^26^. Ethylene response transcription factor plays an important role in signal transduction with disease and stress resistance. Over-expression in plants can improve broad-spectrum resistance of disease and adversity.

Bai, X. *et al*. suggested that atrazine entered the chloroplasts depending on its liposolubility and directly attacked on the electron transport chain, especially PSII, contributing to reactive oxygen species (ROS) burst^27^. Then expression of ROS-related genes and activities of ROS-scavenging enzymes gave an integrative view of physiological state and detoxifying potential under conditions of sensitivity or tolerance. Totally eight up-regulated genes are related to electron transport encoding a series of ROS-scavenging protein in this study, including thioredoxin, glutathione peroxidase (GPX) and glutaredoxin. Thioredoxins have been shown to be involved in supplying reducing power to reductases detoxifying lipid hydroperoxides or repairing oxidized proteins. Furthermore, thioredoxins also acting as regulators of scavenging mechanisms and components of signaling pathways of plant antioxidant network^28^. GPXs function as redox sensors, reducing peroxides and withdrawing electrons from the thiol-based redox transmitters, thereby assisting the system to adjust to the prevailing redox condition of the cell^29^. Glutaredoxin was identified as a good electron donor to atypical type II PRXs. *M. sativa* inoculated has no symptoms such as growth arrest and leaf chlorosis after roots exposed to atrazine. It was suggested that the high expression of electron transport related genes may help to alleviate the detrimental effect of atrazine on electron transport system. Results of these investigations also support the role of atrazine in affecting mitochondrial electron transport and oxidative stress.

We had two hypotheses about the molecular mechanisms of atrazine degradation in soil by AM. One was the secretion of atrazine-degrading enzyme under the stress of atrazine, causing degradation of atrazine in soil; the other was the absorption and degradation of atrazine in the plant. It could be possible that these two mechanisms of atrazine degradation work simultaneously. In agreement with the first hypothesis, our result could be explained by the high expression of laccase. Many studies have shown the laccase has degradation effect on atrazine and many other organic pollutants^30^. The transcriptome data indicate that highly expression of 5 laccase genes under the stress conditions of atrazine. As for the second hypothesis, studies have demonstrated that the atrazine degradation in plants was mainly due to the role of glutathione^31^, forming water-soluble complex conjugate, in addition to the occurrence of hydroxylation and dealkylation. Analysis revealed that highly expression of glutathione peroxidase may play a positive role in atrazine degradation.

## Methods

### Host plant and test fungi

*M. sativa* seeds were scarified with 20 °C to 30 °C in warm water, surface-sterilized with 10% (v/v) solution of hydrogen peroxide for 10 min, then rinsed with sterile distilled water. Seeds were then germinated in the dark on moist filter paper for 24 h. Well germinated seeds were selected for potting experiment.

The inoculum of *G. mosseae* was propagated on sorghum and harvested after 40 days, preserved in the Ecology Laboratory of Heilongjiang University. The inoculum is a mixture of spores, mycelium, sand and root fragments. The inoculum contained about 25 spores per gram.

### Pot experiment design

**Figure 8:**
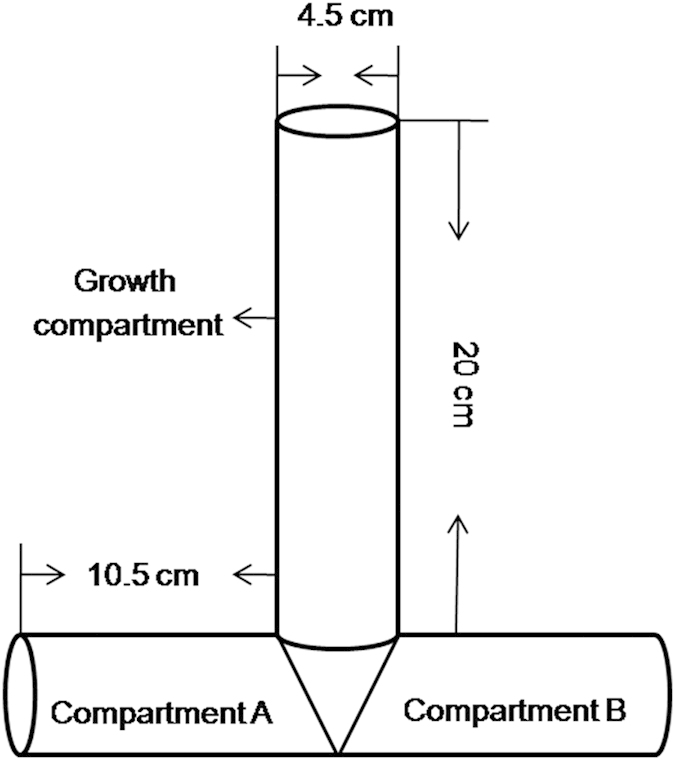
Compartmented cultivation systems.

The compartmented cultivation system partial referred^10^, contains a vertical root compartment-growth compartment (PVC tube, 4.5 cm diameter × 20 cm long) for plant growth and two symmetrical horizontal side-arm compartments (PVC tube, 4.5 cm diameter × 10.5 cm long, compartment A for three atrazine addition levels and compartment B for atrazine-free) (Fig. 8). PVC tubes were surface-sterilized with 0.3% potassium permanganate.

A 5:2:3 (v/v/v) mixture of peat soil, sand and vermiculite was used as growth medium. The medium was autoclaved at 121 °C for 1.5 h, and air dried to moisture content to 20% of water holding capacity (WHC). In the growth compartment, 1.5 kg medium was thoroughly mixed with 30 g inoculum for mycorrhizal treatments and with the same amount of sterilized inoculum (30 g) for non-mycorrhizal treatments. In the side-arm compartments, 200 g atrazine-free medium was added to compartment B, and compartment A was filled with 200 g atrazine-contaminated medium with different addition levels (10, 20 and 30 mg kg^−1^). The experiment was conducted in a controlled-environment growth chamber that maintained a daily 16-h light period. The day/night temperature regime was 25/18 ^o^C. The plants grew for 8 weeks. Deionized water was added as required to maintain soil moisture content at 40% WHC, because semi-arid condition was beneficial to form symbiosis between *M. sativa* and *G. mosseae*.

### Harvesting and sample preparation

To harvest the root samples, the side-arm compartments were separated from the main root compartment after 15 days of culturing. Root samples were carefully washed with tap water, and then rinsed with distilled water, wiped with tissue paper to remove soil particles. Roots were then immediately weighed, wrapped with aluminum foil and snap frozen in liquid nitrogen, and stored at −80 °C. Soil samples were collected from the main root and the side-arm compartments, freeze-dried and stored at −20 °C.

### Root colonization level

Fibrous roots from compartment 1 and compartment 2 were selected to measure the infection rates using an improved acetic acid ink staining method^32^.

### Atrazine degradation rate

For analysis of atrazine, soil from compartments were extracted with n-hexane/acetone mixture (1: 1, v/v) (20 mL /8g soil) using a ultrasonic apparatus for 5 min and centrifuged for 5 min at 2500 rpm. Finally supernatant was air-dried using nitrogen gas. To insure full recovery, soil samples were extracted multiple times. Final product was diluted in 2 mL of methanol. Atrazine content was analyzed by HPLC (Waters, America), using C18 alkyl column (packing: Hypersil ODS2 5 μm, No. E2414789, Dalian Elite Analytical Instruments Co., Ltd). The mobile phase was methanol / water (80:20, v/v). Flow rate was 0.8 mL/min.

### RNA extraction and library preparation for transcriptome analysis

**Figure 9:**
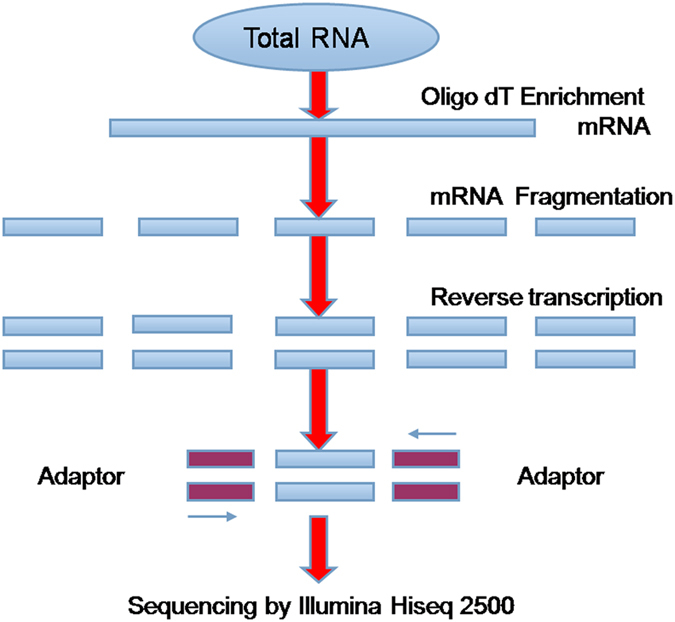
cDNA library construction processes.

Total RNA of roots with and without atrazine were extracted using the TRIzol Reagent (Invitrogen) and treated with DNase I according to manufacturer’s instructions. RNA quality was examined using 2% agarose gel and the concentration was determined using a Nanodrap spectrophotometer (NanoDrop, USA). Then, preparation of cDNA libraries for Illumina HiSeq 2500 sequencing was done using the Truseq^TM^ RNA sample prep kit, following the manufacturer’s instructions. The isolation of mRNA, fragment interruption, cDNA synthesis, addition of adapters, PCR amplification and RNASeq were performed by staff at Shanghai Majorbio Bio-Pharm Technology (Shanghai, China). Poly-A mRNA was isolated using Magnetic Oligo (dT) Beads, and then broken into small pieces using divalent cations under an elevated temperature. Then the double-stranded cDNA was synthesized using the SuperScript Double-Stranded cDNA Synthesis kit (Invitrogen, Camarillo, CA) with random hexamer primers (Illumina). The synthesized cDNA was subjected to end-repair and phosphorylation using T4 DNA polymerase, Klenow DNA polymerase and T4 PNK. These repaired cDNA fragments were 3′ adenylated using Klenow Exo- (3′ to 5′ exo minus, Illumina). Illumina Paired-end adapters were ligated to the ends of these 3′-adenylated cDNA fragments. Fragments (200 bp ± 25 bp) were then separated by agarose gel electrophoresis and selected for PCR amplification as sequencing templates. Finally, the mRNA-seq library was constructed for sequencing on the Illumina HiSeqTM 2500 sequencing platform. cDNA library construction process was shown in Fig. 9.

### De novo assembly and annotation

Transcriptome de novo assembly was carried out using Trinity Software. In order to reduce redundancy and chimeras in the Trinity pipeline, we used CAP3 tomerge and combine highly similarly assembled sequences into unisequences.

Sequences were annotated using a series of sequential BLAST searches designed to find the most descriptive annotation for each sequence. Assembled unique transcripts were compared with sequences in Nr using the BLAST algorithm, the GI accessions of best hits were retrieved, and the GO accessions were mapped to GO terms according to molecular function, biological process, and cellular component ontologies (http://www.geneontology.org/). The remaining sequences that putatively encoded proteins were searched against the SwissProt protein database (http://www.expasy.ch/sprot), the KEGG pathway database, and the COG database (http://www.ncbi.nlm.nih.gov/COG) by applying a typical E-value threshold of less than 10^−5^. Moreover, the edgeR (http://www.bioconductor.org/packages/release/bioc/html/edgeR.html) tool was applied in order to calculate the different expression and the significant differences.

### Validation of differentially expressed genes (RT-PCR test)

The RT-PCR is design to exclude the possibility of false high throughput mRNA sequencing. Hence, 10 differentially expressed genes closely associated were chosen for the experimentation. The primer sequences are shown in Table 4, the amplification reaction system and procedure of PCR are shown in Tables 5 and 6.

## Additional Information

**How to cite this article**: Song, F. *et al*. Transcriptome analysis of *Glomus mosseae/Medicago sativa* mycorrhiza on atrazine stress. *Sci. Rep.* **6**, 20245; doi: 10.1038/srep20245 (2016).

## Figures and Tables

**Table 1:** Summary of data generated for *G. mosseae* and *M. sativa* transcriptome.

Total number of reads	103134502
Total clean nucleotides (bp)	10416584702
Total isogenes	75957
Average length of all contigs	1132.66
Range of contig length (bp)	351–14643
Number of total genes	33948
Length of all residues (bp)	86033596

**Table 2:** Anti-stress/anti-disease protein of *M. sativa*.

**No. of genes**	**Length (bp)**	**Accession number**	**NR tophit description**	**Identity (%)**	**log2FC1 (ATa/AT)**
comp42025_c0	91	gi|357475395	Ethylene responsive transcription factor 2b [*Medicago truncatula*]	71	8.89
comp68957_c0	138	gi|357498565	Kunitz-type trypsin inhibitor-like 1 protein [*Medicago truncatula*]	81	8.82
comp53515_c0	59	gi|357473133	BURP domain-containing protein [*Medicago truncatula*]	90	8.55
comp211111_c0	1059	gi|193237563	transcription factor C2H2 [*Lotus japonicus*]	68	8.30
comp29588_c0	53	gi|357469835	F-box/LRR-repeat protein [*Medicago truncatula*]	95	7.94
comp341394_c0	299	gi|357453133	CCR4 associated factor 1-related protein [*Medicago truncatula*]	84	7.26
comp32566_c0	151	gi|357452821	Disease-resistance protein [*Medicago truncatula*]	69	6.39
comp279364_c0	160	gi|357443099	NAC domain protein [*Medicago truncatula*]	91	6.31
comp350759_c0	133	gi|357455019	F-box/kelch-repeat protein [*Medicago truncatula*]	92	5.85
comp49732_c0	119	gi|357497861	Kunitz-type serine protease inhibitor DrTI [*Medicago truncatula*]	69	5.85
comp321001_c0	349	gi|357515301	Flavonol sulfotransferase-like protein [*Medicago truncatula*]	84	4.99
comp347025_c0	222	gi|357511791	Wall-associated receptor kinase-like protein [*Medicago truncatula*]	98	4.86
comp229988_c0	523	gi|357499349	Resistance gene analog protein [*Medicago truncatula*]	100	4.23
comp68237_c0	188	gi|357498545	Kunitz-type trypsin inhibitor-like 2 protein [*Medicago truncatula*]	61	4.23
comp60043_c0	96	gi|357512199	Benzoyl coenzyme A benzyl alcohol benzoyl transferase [*Medicago truncatula*]	99	4
comp331723_c0	182	gi|357515301	Flavonol sulfotransferase-like protein [*Medicago truncatula*]	95	3.87
comp28527_c0	125	gi|357503357	Heat shock protein [*Medicago truncatula*]	73	7.59
comp60376_c0	153	gi|357499615	Disease resistance-like protein [*Medicago truncatula*]	61	3.11
comp67472_c0	137	gi|357498545	Kunitz-type trypsin inhibitor-like 2 protein [*Medicago truncatula*]	35	3.02
comp54873_c0	298	gi|357466893	Receptor-like protein kinase [*Medicago truncatula*]	99	3.02
comp59808_c0	73	gi|357441047	Inhibitor of trypsin and hageman factor [*Medicago truncatula*]	75	2.88
comp22103_c0	372	gi|357473133	BURP domain-containing protein [*Medicago truncatula*]	95	2.85
comp72039_c0	215	gi|71534908	BURP domain-containing protein, partial [*Medicago sativa*]	96	2.76
comp68035_c0	158	gi|357468219	Germin-like protein [*Medicago truncatula*]	98	2.71
comp67862_c0	418	gi|357438305	NBS-containing resistance-like protein [*Medicago truncatula*]	65	2.71
comp70036_c0	614	gi|357437677	Snakin-1 [*Medicago truncatula*]	100	2.63
comp68404_c0	214	gi|357497861	Kunitz-type serine protease inhibitor DrTI [*Medicago truncatula*]	65	2.36
comp77797_c0	872	gi|357493453	Receptor-like protein kinase [*Medicago truncatula*]	87	2.28
comp78808_c0	494	gi|357492309	ER glycerol-phosphate acyltransferase [*Medicago truncatula*]	98	2.08
comp74170_c0	591	gi|357476949	Monocopper oxidase-like protein SKU5 [*Medicago truncatula*]	99	2.03
comp75799_c0	320	gi|357481947	Knolle [*Medicago truncatula*]	99	2.01
comp67429_c0	153	gi|357497581	Anthocyanidin 3-O-glucosyltransferase [*Medicago truncatula*]	98	7.09
comp33808_c0	370	gi|30686851	Sulfite exporter TauE/SafE family protein [*Arabidopsis thaliana*]	100	5.74
comp35862_c0	255	gi|357477405	Nitrate transporter (NTL1) [*Medicago truncatula*]	98	4.61
comp65594_c0	114	gi|357497581	Anthocyanidin 3-O-glucosyltransferase [*Medicago truncatula*]	99	4.42
comp76446_c0	183	gi|357480825	Early nodulin-like protein [*Medicago truncatula*]	99	2.45
comp47317_c0	826	gi|357440947	CCP [*Medicago truncatula*]	91	2.26
comp67605_c0	252	gi|357509773	CCP-like protein [*Medicago truncatula*]	95	2.36
comp75321_c0	309	gi|357518019	Thaumatin-like protein [*Medicago truncatula*]	99	2.25

**Table 3:** Laccase, electron transport-related protein and zinc finger protein of *M. sativa* (partial listed).

**No. of genes**	**Length (bp)**	**Accession number**	**NR tophit description**	**Identity (%)**	**log2FC1 (ATa/AT)**
Laccase					
comp80087_c0	568	gi|357492827	Laccase [*Medicago truncatula*]	100	2.24
comp65604_c0	342	gi|357491147	Laccase-like multicopper oxidase [*Medicago truncatula*]	100	2.26
comp81470_c0	569	gi|357490575	Laccase 1a [*Medicago truncatula*]	98	2.26
comp57797_c0	249	gi|357505329	Laccase [*Medicago truncatula*]	98	2.85
comp85523_c0	581	gi|357483501	Laccase-11 [*Medicago truncatula*]	98	2.82
Electron transport-related protein					
comp67947_c0	144	gi|357486521	Thioredoxin [Medicago *truncatula*]	99	2.73
comp72639_c0	828	gi|357445481	Potassium channel [*Medicago truncatula*]	98	2.06
comp74170_c0	591	gi|357476949	Monocopper oxidase-like protein SKU5 [*Medicago truncatula*]	99	2.03
comp29448_c0	138	gi|124359194	Na+/H+ antiporter-like protein, putative [*Medicago truncatula*]	89	6.46
comp59009_c0	116	gi|357511171	Monothiol glutaredoxin-S6 [*Medicago truncatula*]	100	7.30
comp441_c0	69	gi|357520459	Glutathione peroxidase [*Medicago truncatula*]	93	5.95
comp72359_c0	76	gi|269315890	thioredoxin h7 [*Medicago truncatula*]	100	2.63
comp50160_c0	121	gi|357511173	Glutaredoxin [*Medicago truncatula*]	98	2.43
Zinc finger protein					
comp23376_c0	145	gi|357479803	CONSTANS-like zinc finger protein [*Medicago truncatula*]	98	6.66
comp13165_c0	98	gi|357462041	Zinc finger CCCH domain-containing protein [*Medicago truncatula*]	96	6.46
comp63527_c0	136	gi|357457663	Zinc finger A20 and AN1 domain-containing stress-associated protein [*Medicago truncatula*]	77	5.85
comp204408_c0	228	gi|357452119	Zinc finger C2H2 type family protein [*Medicago truncatula*]	83	4.79
comp62891_c0	265	gi|358348823	Zinc finger CCCH domain-containing protein, partial [*Medicago truncatula*]	100	2.38

**Table 4:** The primer sequences.

**Gene number**	**Primer 1**	**Primer 2**
comp441_c0	GATGGTATGGGAAATGA	TTGGTTCCAGGTTCTTC
comp80087_c0	ATTGGCATGGAGTTAGA	TTGGCTTAGTGAAAGGA
comp81470_c0	TTCTACTAGCTGCTTATTG	ATCTTGTGGCATTCTTC
comp57797_c0	CTTTCACAATGCCAACC	TGCATCACAAGCTCCAC
comp29448_c0	AGCCTACAAAGCCAGTG	ATGACCAGGCTTCTTAC
comp74170_c0	CCACGAAGCCTCTAACC	ACGAGCTATTCCATCCC
comp13165_c0	TCTGTGGCTCATAGTGG	GAACTAGGTTTGTTCTCCC
comp63527_c0	CAACGACAATCTTACCACCTT	AATCCAGCCAACCCAAC
comp50160_c0	GCAAAAGCACTTGTCCC	TACCACCTATGATGTTGTC
comp279364_c0	AAGAATATGTGGTGGTAAAG	CTGTTGCTGCTGGTAAA

**Table 5:** The amplification reaction system of PCR.

**Components**	**Volume**
10× PCR buffer (Mg^2+^ Free)	2.5 μL
50× Advantage cDNA polymerase	0.5 μL
Mg^2+^(25 mM)	1.5 μL
dNTP (10 μM)	0.5 μL
Primer 1 (10 μM)	1.0 μL
Primer 2 (10 μM)	1.0 μL
Sterile H_2_O	18.0 μL
Total volume	25 μL

**Table 6:** The amplification reaction procedure of PCR.

**Steps**	**Temperature**	**Time**
Step 1	94 °C	10 min
Step 2	94 °C	1 min
Step 3	54 °C, 50 °C⋇	30 sec
